# Age and antibiotic use influence longitudinal dynamics of the upper respiratory microbiome in children with recurrent acute otitis media

**DOI:** 10.1128/msphere.00468-25

**Published:** 2025-11-12

**Authors:** Jillian H. Hurst, Yue Xing, Qunfeng Dong, Alejandro Hoberman, Xiang Gao, Nader Shaikh

**Affiliations:** 1 Department of Pediatrics, Division of Infectious Diseases, Duke University School of Medicine 12277 , Durham, North Carolina, USA; 2 Duke Microbiome Center, Duke University School of Medicine 12277 , Durham, North Carolina, USA; 3 Department of Medicine, Stritch School of Medicine, Loyola University Chicago 2456, Chicago, Illinois, USA; 4 Division of General Academic Pediatrics, University of Pittsburgh School of Medicine, Children’s Hospital of Pittsburgh 12317 , Pittsburgh, Pennsylvania, USA; Medical College of Wisconsin, Milwaukee, Wisconsin, USA

**Keywords:** AOM, 16S rRNA sequencing, antimicrobials, antibiotics, resistance

## Abstract

The upper respiratory tract (URT) microbiome has emerged as a key component of acute otitis media (AOM) pathophysiology; however, few studies conducted to date have evaluated URT microbiome composition in children with recurrent AOM (rAOM). We collected serial nasopharyngeal samples from a cohort of 58 children, 6 to 35 months of age, over a one-year period. Samples were analyzed using 16S rRNA sequencing and PCR-based assays for common otopathogens and respiratory viruses. Age was strongly associated with differential abundance of specific genera, including increased abundance of genera associated with respiratory health (e.g., *Dolosigranulum*, *Corynebacterium*). In contrast, samples collected during AOM episodes or within 30 days of receipt of an antibiotic had a lower relative abundance of these genera. Further, the number of antibiotic-free days prior to sample collection was associated with global changes in microbiome composition. Unsupervised clustering identified three microbiome profiles that differed by incidence of AOM, bacterial otopathogen burden, symptom score, and number of antibiotic-free days prior to sample collection. Increasing age was associated with transition to profiles characterized by lower incidence of AOM and bacterial otopathogen burden, while antibiotic use was associated with transition to a profile associated with greater incidence of AOM. Our findings indicate that alterations of the microbiome associated with aging may contribute to decreased incidence of AOM as children age, while systemic antibiotic use may induce dysbiosis, thereby enhancing AOM susceptibility.

**IMPORTANCE:**

Ear infections are the most common bacterial infection among young children and the leading cause of healthcare visits and antibiotic prescriptions. This study explores the connection between the microbiome of the nose—the community of microorganisms that live in different areas of the human body—and recurrent ear infections in young children. An analysis of nasal swabs collected from 58 children over a year showed that as children age, they tend to have fewer bacterial pathogens and more species that are associated with a healthy state in their microbiomes. These more mature microbiomes were associated with fewer ear infections. In contrast, recent use of antibiotics was associated with microbiomes that had more bacterial pathogens and that were associated with greater ear infection incidence. Overall, these findings indicate that the microbiome may be a key factor in reduced ear infections as children age.

## INTRODUCTION

Acute otitis media (AOM; middle ear infection) is the most common bacterial infection of childhood and the leading cause of pediatric health care visits and antibiotic prescriptions worldwide (1). There are estimated to be more than 11,000,000 cases of AOM in the United States annually, with the majority of cases among children under 3 years of age (2). AOM is the most common reason for antibiotic receipt in children, resulting in over 10,000,000 antibiotic prescriptions and 30,000,000 medical visits each year (2–5). While most children have only a few AOM episodes in the first few years of life, an estimated 5–30% of children develop recurrent AOM (rAOM), defined as three or more episodes in a 6-month period or four or more episodes in a year (6, 7). These AOM-prone children typically receive repeated courses of antibiotics, and a substantial proportion undergo surgery for tympanostomy tube placement, despite its questionable efficacy (8, 9). Though rAOM represents a substantial burden in terms of healthcare utilization and quality of life, only a few studies have evaluated the underlying microbiology of the disease.

There is accumulating evidence that the microbiome of the upper respiratory tract (URT) is a key mediator of susceptibility to acute respiratory illnesses, including AOM. To date, the majority of studies evaluating the relationship between the microbiome and AOM have focused on the abundance of *Streptococcus pneumoniae*, *Moraxella catarrhalis*, and non-typeable *Haemophilus influenzae*, three respiratory pathobionts that are thought to underlie up to 95% of AOM episodes (10–12). Colonization with these pathobionts early in infancy has been associated with increased risk of AOM and other respiratory infections later in childhood (13–15). In contrast, increased abundance of the respiratory commensal genera *Corynebacterium* and *Dolosigranulum* has been associated with reduced risk of AOM and fewer acute respiratory infections (ARIs) (16–18). Taken together, these data indicate that AOM occurs in conjunction with respiratory dysbiosis and that alterations in the respiratory microbiome may indicate which children will be susceptible to future AOM episodes and other ARIs (19–21).

Currently, limited data exist regarding longitudinal microbiome composition in rAOM (14, 22, 23). Cross-sectional case-control studies of children with rAOM have identified decreased diversity within the nasopharyngeal microbiome and reduced abundances of *Corynebacterium* and *Dolosigranulum* species compared with those in healthy children (16, 24). In addition to changes in bacterial composition, children with rAOM have increased abundance of viral pathogens, including enterovirus/human rhinovirus, parainfluenza, and respiratory syncytial virus (RSV) (25–27). An evaluation of longitudinal microbiome changes in this population could help identify children who may be at risk of additional AOM episodes and help elucidate the relationship between antibiotic exposures, tympanostomy tube placement, and the URT microbiome. We evaluated microbiome composition in serial nasopharyngeal swabs collected over one year from a cohort of 58 children 6 to 35 months of age who had been previously diagnosed with rAOM (9). Using these data, we assessed for associations between microbiome composition and the development of AOM/otorrhea. We further assessed for exposures associated with alterations in microbiome composition, including antibiotic receipt and placement of tympanostomy tubes.

## MATERIALS AND METHODS

### Participant eligibility and recruitment

The current study was performed using samples and data from 250 children aged 6 to 35 months with rAOM, who were prospectively enrolled in a randomized study comparing the efficacy of tympanostomy tubes vs medical management in reducing future episodes of AOM, as previously described (9). During the two-year follow-up period, children were routinely sampled using nasopharyngeal swabs (i) every 16 weeks, (ii) each time respiratory symptoms were present for at least five days, (iii) if symptoms suggestive of AOM were reported, and (iv) if the child was diagnosed with AOM (defined as unilateral or bilateral tympanic membrane bulging or tympanostomy tube otorrhea; hereafter referred to as AOM). Children enrolled were well characterized clinically, with all assessments conducted by study clinicians who had participated in an otoscopic validation program. Sociodemographic data were captured for each participant at enrollment, including age, sex, race, ethnicity, the highest level of education completed by the child’s parent/primary caregiver, the presence of a smoker in the child’s home, and the type of health insurance. At each study visit, the following data were recorded: age, season, days since enrollment, exposure to other children for 10 or more hours per week, the presence or absence of AOM/otorrhea, and AOM-SOS score (28). Records were also kept of the dates of antibiotic treatment or surgeries (i.e., tympanostomy tube placement), with antibiotic exposures defined as the number of days between the date of last dose of antibiotics and the date of sample collection (i.e., antibiotic-free days). The current study includes 305 samples from the first 58 consecutive children enrolled during their first year of follow-up.

### Specimen collection and processing

Samples were collected as previously described (9). Briefly, nasopharyngeal samples were collected with flocked swabs (Copan, Murrieta, CA), and liquid Amies medium (Copan, Murrieta, CA) was used for transport. Samples were typically transported to the lab within 1 h of collection but were stored at 4°C if delays in transport were anticipated. Upon arrival at the processing laboratory, samples were vortexed, divided into four aliquots, and stored at −80°C. Aliquots were only thawed a single time for use in the assays described below. Swabs were cultured for *S. pneumoniae*, *M. catarrhalis*, and *H. influenzae* on the day of sample collection (see below).

### 16S rRNA gene amplicon sequencing and analysis

An aliquot with no added preservatives was used for 16S rRNA gene amplicon sequencing. The aliquot was shipped on dry ice to the Environmental Sample Preparation and Sequencing Facility at Argonne National Laboratory (Lemont, IL) for 16S rRNA gene amplicon analysis. DNA was extracted using the 96-well format DNEasy PowerSoil kit (Qiagen, Germantown, MD) according to the manufacturer’s instructions. For 16S rRNA gene amplicon sequencing, the V4 region of the 16S rRNA gene (515F-806R) was amplified using PCR with a 12-base barcode on the forward primer (forward primer: 5′-GTG**Y**CAGCMGCCGCGGTAA-3′; reverse primer: 5′-GGACTAC**N**VGGGTWTCTAAT-3′) (29). The PCR mixture contained 9.5 µL of PCR water, 12.5 µL of Quantabio’s AccuStart II PCR ToughMix (Quantabio, Beverly, MA; 2× concentration, 1× final), 1 µL of Golay barcode-tagged forward primer (5  µM concentration, 200 pM final), 1 µL reverse primer (5  µM concentration, 200 pM final), and 1 µL of template DNA. The PCR conditions were set to 94°C for 3 min to denature the DNA, with 35 cycles at 94°C for 45 s, 50°C for 60 s, and 72°C for 90 s, and a final extension of 10 min at 72°C. Samples were pooled into a single tube, quantified, and diluted to 2 nM, and 6.75 pM of 10% PhiX (Illumina, San Diego, CA) was added. Amplicons were sequenced on a 151 bp by 12 bp by 151 bp Illumina MiSeq run. Negative controls were incorporated at all stages through sequencing, including an unused flocked swab and medium aliquot. A positive control of *Pseudomonas aeruginosa* strain PA14 was also included through all stages of sample preparation and sequencing. Of the 50,059 sequences recovered for this positive control, 49,760 (99.4%) were assigned to *Pseudomonas*. The remaining sequences in this sample were assigned to a mixture of taxa consisting of *Moraxella* (0.03%), *Parabacteroides* (0.06%), *Turicibacter* (0.02%), and *Mucispirillum* (0.01%), with the remainder unassigned. Sequencing reads were analyzed through a pipeline using DADA2 1.26.0 (30). Taxonomic assignments of amplicon sequence variants (ASVs) were made based on alignment with the expanded Human Oral Microbiome Database version 15.1 (31). Presumed reagent contaminant ASVs were removed using the frequency method in the *decontam* R package version 3.20 (threshold = 0.10) (32). Three samples with <1,000 sequencing reads after quality filtering were excluded. The median (IQR) reads per sample were 17,141 (11,083–22,406) prior to filtering and 16,723 (10,752–21,763) after filtering. There were 810 unique ASVs after quality filtering, which were assigned to 40 bacterial genera and six phyla.

### Bacterial identification

For bacterial culture, the swabs were used to inoculate three types of media: trypticase soy agar, trypticase soy agar with 5% sheep blood, and chocolate agar for the culture of *S. pneumoniae*, *H. influenzae*, and *M. catarrhalis*, respectively. Cultures were incubated overnight at 37°C with 5% CO_2_. If no growth was present after overnight incubation, the culture was re-incubated for another 24 h. Growth of pathogenic bacteria (*S. pneumoniae*, *H. influenzae*, and *M. catarrhalis*) was assessed using standard techniques (i.e., four quadrant streak; growth in 0, 1, 2, 3, or 4 quadrants); 3+ and 4+ were considered heavy growth (33). Culture identification was performed using the Vitek 2 (Biomérieux, Durham, NC).

### Viral identification

Nucleic acid extraction was performed with the ABI MagMax 96 Express automated instrument and the MagMax Viral Isolation Kit (Applied Biosystems, Foster City, CA) from one of the four aliquots prepared from the original sample. Individual real-time RT-PCR assays for the detection of human adenovirus (HAdV), influenza A/B/C, human metapneumovirus (HMPV), enterovirus/human rhinovirus (EV-HRV), parainfluenza virus (PIV) subtypes 1–4, and respiratory syncytial virus (RSV) were performed as previously described (34–40). All specimens were tested for RNase P to confirm RNA integrity and monitor for PCR inhibitors. Samples were run in batches on an ABI Step One Plus 96-well real-time PCR instrument (Thermo Fisher Scientific, Waltham, MA). Assays were performed with 25 µL reaction mixtures containing 5 µL of extracted RNA using an ABI StepOnePlus real-time PCR system (Applied Biosystems, Waltham, MA) and the AgPath-ID One-Step RT-PCR kit (Applied Biosystems, Waltham, MA). Negative and positive controls were included with each run. Specimens were considered positive if the cycle threshold (Ct) value was <40 cycles.

### Statistical analyses

We evaluated associations between participant and sample characteristics and AOM status or antibiotic exposures using chi-square (χ²) or Fisher’s exact tests (categorical variables) and Wilcoxon rank-sum or Kruskal-Wallis tests (continuous variables). ANOVA was used to evaluate for differences in alpha diversity across groups. We evaluated microbiome diversity and stability by calculating the alpha (Shannon index and observed ASVs) and beta diversity (Euclidean distance) using the vegan R package version 2.7-0 (41). The microbiome R package version 1.8.0 was used to generate centered log-ratio (CLR)–transformed sample counts to evaluate between-sample compositional differences (42, 43). We used ANOVA tests to compare microbiome alpha diversity across groups with different exposures. We compared Euclidean distance with PERMANOVA using the adonis2 function within the vegan R package version 2.7-0 (44) with subject as a repeated measurement. We used lmer and glmer linear mixed models (R package lme4 version 1.1-35.3) to evaluate associations between sociodemographic factors and exposures and the relative abundances of bacterial genera within the participant microbiome samples (45). These analyses included sex, participation in congregate care settings, age, AOM status, antibiotic exposures, respiratory virus detection, presence of tympanostomy tubes, smoker in household, and presence of a bacterial pathogen as exposures. A linear mixed-effects model was used to assess for correlations between the relative abundance of specific genera within participant nasopharyngeal samples and antibiotic-free days, adjusted for covariates (age at time of sample collection, smoker in child’s home, season of sample collection, presence of bacterial and viral pathogens, and AOM-SOS score). Analyses were limited to bacterial genera present in at least 10% of nasopharyngeal samples. The comparisons were corrected for the false discovery rate using the Benjamini–Hochberg procedure, and a *q* value threshold for significance of 0.20 was used. The models included subject as a random effect. We used k-medoids clustering and the Calinski-Harabasz index to classify samples into distinct nasopharyngeal microbiome profiles. A logistic mixed-effects model was used to evaluate for associations between microbiome cluster transitions and antibiotic receipt, age, and tympanostomy tube placement, which were tested simultaneously within a single model.

## RESULTS

### Study population

The study population included 58 children, 22 (38%) of whom were female, 41 (71%) who were non-white, and 9 (16%) who were Hispanic. Most children (*n* = 42, 72%) did not reside in a household with someone who smoked, and 47 (81%) spent more than 10 h a week in a congregate care setting (i.e., with three or more other children). A total of 305 samples were included in this study, and the children had a median (interquartile range [IQR]) of 5 (4–6.8) samples collected, with a median (IQR) of 103 days (58, 131) between samples (Table 1). Of the 305 visits at which samples were collected, 105 (34%) occurred during an AOM episode, with a median (IQR) of 1 (1–3) AOM visits per child. The children had a median (IQR) of 1 (0–2) visit that included receipt of antibiotics.

**TABLE 1:** Characteristics of the study population

Participant characteristic (*n* = 58)	Result
Age (months), median (IQR) at enrollment	13.5 (11.0, 19.8)
Female sex, *n* (%)	22 (38%)
Race, *n* (%)	
Black/African American	36 (62%)
White	17 (29%)
Other race	5 (9%)
Hispanic ethnicity, *n* (%)	9 (16%)
Maternal education, *n* (%)	
Less than high school	1 (2%)
High school graduate or GED	15 (26%)
Technical school/some college/associates degree	24 (41%)
College graduate	9 (16%)
Post-graduate	9 (16%)
Smoker in child’s home, *n* (%)	16 (28%)
Insurance type, *n* (%)	
Private	20 (35%)
Public	37 (64%)
None	1 (2%)
Exposed to three or more children for 10 + hours/week, *n* (%)	47 (81%)
Tympanostomy tubes placed during the study	33 (57%)
Visits/samples per child, median (IQR)	5.0 (4.0, 6.8)
Days between visits/samples, median (IQR)	103 (58, 131)
AOM/otorrhea visits per child, median (IQR)	1.0 (1.0, 3.0)
Visits at which systemic antibiotics were prescribed, median (IQR)	1.0 (0.0, 2.0)
Visits occurring after tympanostomy tube placement, *n* (%)	144 (47%)

### Associations between AOM, sociodemographic characteristics, and respiratory pathogen detection on culture

We first sought to identify associations between AOM and sociodemographic and clinical characteristics (Table 2). Children were younger at visits where AOM was identified compared to at visits where AOM was not detected (18 months versus 24 months, *P* = 2 × 10^−4^). Visits with AOM were more likely to occur during colder months (*P* = 0.02). Further, we found that the prevalence of respiratory and viral pathogens detected in these samples varied by the season of collection (Table S1). The majority of viral pathogens were most frequently detected in January–April, while parainfluenza was more prevalent during May–September and October–November. EV-HRV was highly prevalent regardless of season, with 56–67% of samples testing positive for the virus. Among bacterial respiratory pathogens, GAS was the least prevalent across all three periods, with a detection rate of only 10% during January–April. *M. catarrhalis* was most prevalent in the colder months, with a prevalence of 67% in January–April and October–December. *H. influenzae* was most frequently detected in samples collected in January–April (54%), while *S. pneumoniae* was present in approximately half of all samples regardless of season. AOM was detected at approximately the same rate regardless of whether children had tympanostomy tubes at a given visit. There were fewer antibiotic-free days prior to AOM visits compared to AOM-free visits (55.5 days vs 124 days, *P* < 1 × 10^−4^).

**TABLE 2:** Clinical and microbiome characteristics by AOM status

Sample characteristic	No AOM or otorrhea (*n* = 173, 64.6%)	AOM or otorrhea present (*n* = 95, 35.4%)	*P*^*a*^
Age (months), median (IQR)	24 (17, 30)	18 (13.5, 24.5)	**2 × 10^−4^**
Season			
January through April	42 (24.3%)	36 (37.9%)	**0.02**
May through September	86 (49.7%)	32 (33.7%)	
October through December	45 (26%)	27 (28.4%)	
Tympanostomy tubes placed, *n* (%)	85 (49.1%)	44 (46.3%)	0.75
Antibiotic-free days, median (IQR)	124 (55, 298)	55.5 (22.5, 132.5)	**<1 × 10^−4^**
Bacterial pathogens detected (any)	133 (76.9%)	88 (92.6%)	**0.002**
*S. pneumoniae*	75 (43.4%)	57 (60%)	**0.013**
*H. influenzae*	68 (39.3%)	52 (54.7%)	**0.021**
*M. catarrhalis*	94 (54.3%)	64 (67.4%)	0.052
Number of bacterial pathogens detected			**0.002**
0	40 (23.1%)	7 (7.4%)	
1	51 (29.5%)	27 (28.4%)	
2	60 (34.7%)	37 (38.9%)	
3	22 (12.7%)	24 (25.3%)	
Respiratory viruses detected, *n* (%)	129 (74.6%)	84 (88.4%)	**0.012**
Influenza A/B/C	11 (6.4%)	5 (5.3%)	0.926
Metapneumovirus	14 (8.1%)	12 (12.6%)	0.325
RSV	11 (6.4%)	13 (13.7%)	0.074
Enterovirus/human rhinovirus	110 (63.6%)	54 (56.8%)	0.341
Parainfluenza virus	24 (13.9%)	21 (22.1%)	0.120
Human adenovirus	44 (25.4%)	32 (33.7%)	0.196
Number of viruses detected, median (IQR)	1 (0, 2)	1 (1, 2)	0.0498
Shannon diversity, median (IQR)	1.17 (0.74, 1.56)	1.14 (0.81, 1.53)	0.545
Microbiome richness: number of unique ASVs, median (IQR)	15 (11, 22)	14 (11, 18)	0.084

^*a*^ Bold values indicate *P *< 0.05.

We next assessed for differences in the detection of bacterial and viral respiratory pathogens in samples collected AOM versus AOM-free visits (Table 2). While *S. pneumoniae*, *M. catarrhalis*, and *H. influenzae* were highly prevalent regardless of AOM status, *S. pneumoniae* and *H. influenzae* were more prevalent in samples collected at AOM visits compared to samples collected during AOM-free visits (*S. pneumoniae:* 92.6% vs 76.9%, *P* = 0.013; *H. influenzae:* 60% vs 43.4%, *P* = 0.021). We did not detect a significant difference in the prevalence of *M. catarrhalis*. Notably, the number of bacterial pathogens identified was greater in samples collected during AOM visits compared to samples collected at AOM-free visits (*P* = 0.002). We did not detect any bacterial pathogens in nearly one quarter (23.1% [40/173]) of samples collected during an AOM-free visit. Comparatively, at least one bacterial pathogen was detected in all but 7.4% (7/95) samples collected during an AOM visit. All three pathogens were detected in 25.3% (24/95) of samples collected during an AOM visit compared with 12.7% (22/173) of samples collected at an AOM-free visit. Samples collected during an AOM visit were slightly more likely than samples collected during an AOM-free visit to have at least one viral pathogen detected (88.4% [84/95] vs 74.6% [129/173]; *P* = 0.012). However, we did not identify any significant differences in the detection of specific respiratory viruses.

### Microbiome features associated with AOM status and antibiotic exposures

**Fig 1:**
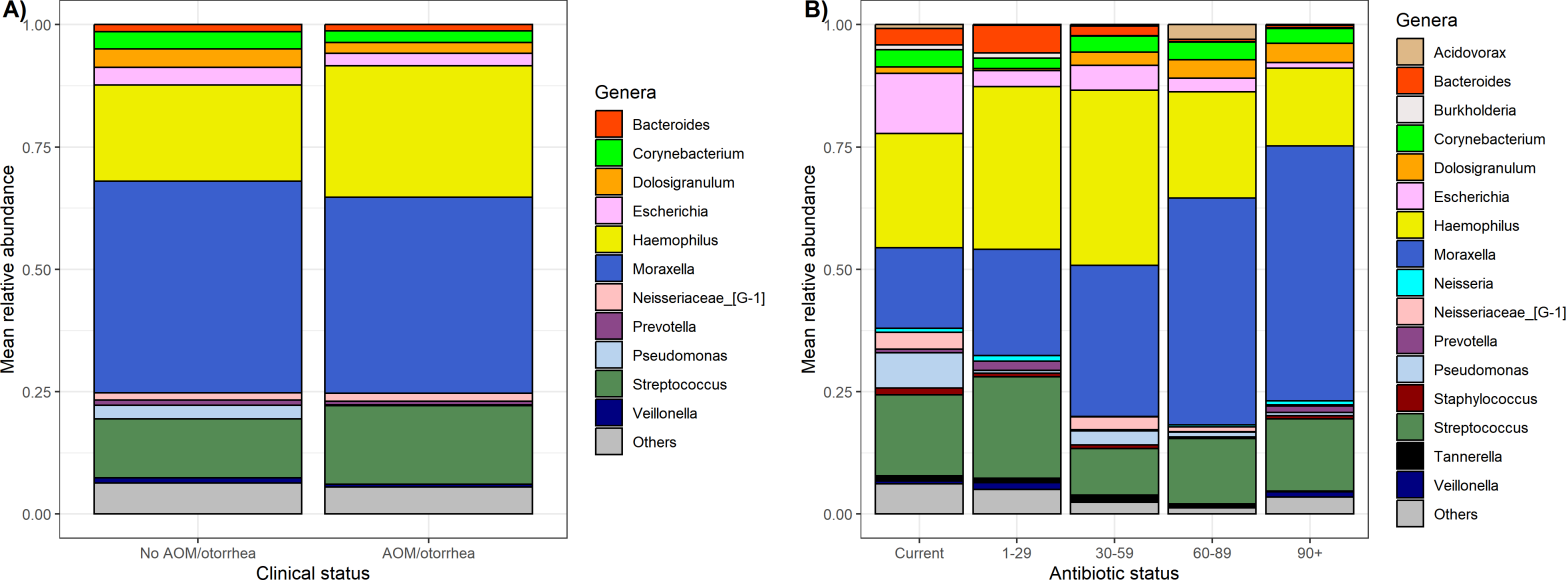
Relative abundances of highly abundant bacterial genera by AOM/otorrhea status and days since antibiotic receipt. (**A**) Each bar depicts the mean relative abundance of highly abundant genera in nasopharyngeal samples collected from participants who were classified by the presence or absence of AOM/otorrhea. (**B**) Each bar depicts the mean relative abundance of highly abundant genera in nasopharyngeal samples collected from participants classified by the number of antibiotic-free days, evaluated as a categorical variable. Of note, participants classified as currently receiving antibiotics (first bar) had been taking antibiotics for a median of 7 days (IQR: 4, 9 days).

We next evaluated for differences in overall microbiome composition by AOM status. We did not detect any differences in Shannon diversity or richness between samples collected at AOM visits and AOM-free visits (Table 2); however, we noted significant differences in the prevalence of the most abundant genera after adjusting for age at the time of sample collection, presence of a smoker in the child’s home, participation in congregate care settings, season of sample collection, presence of bacterial and viral pathogens, and AOM-SOS score (Fig. 1A, *P* = 0.001). Samples collected during AOM-free visits had greater relative abundances of *Corynebacterium*, *Dolosigranulum*, and *Moraxella*, while samples collected during AOM visits had greater relative abundances of *Haemophilus* and *Streptococcus*. Because AOM visits were preceded by fewer antibiotic-free days compared to AOM-free visits, we hypothesized that microbiome composition would be associated with the number of days since antibiotic receipt. Samples were grouped by AOM-free days, including currently taking antibiotics, <30 days, 30–59 days, 60–89 days, and 90 or more antibiotic-free days (Fig. 1B). After adjusting for the covariates described above, we found that the number of antibiotic-free days was significantly associated with overall microbiome composition (*P* = 0.001). Further, the relative abundance of *Corynebacterium*, *Dolosigranulum*, and *Moraxella* ASVs was positively correlated with the number of antibiotic-free days preceding sample collection (*Corynebacterium*: ρ = 0.26, *P* = 1 × 10^−4^; *Dolosigranulum*: ρ = 0.32, *P* < 1 × 10^−4^; *Moraxella*: ρ = 0.36, *P* < 1 × 10^−4^; Table S2). We did not identify any significant associations with Shannon diversity or richness (Fig. S1).

In order to evaluate for associations between microbiome composition and antibiotic-free days prior to sample collection, we used a mixed effects linear regression model to identify specific changes in microbiome composition associated with time since receipt of an antibiotic, adjusting for the following factors at the time of sample collection: AOM status, participation in congregate care settings, smoker in the child’s home, season, presence of tympanostomy tubes, age, AOM-SOS score, and detection of bacterial and viral respiratory pathogens (Table S2 and S3). The relative abundance of *Moraxella* ASVs was significantly greater in samples collected more than 90 days after antibiotic receipt compared to samples collected less than 30 days after antibiotic receipt (estimate: 2.4108, *P* = 0.0044). In contrast, the relative abundance of *Escherichia* ASVs was significantly lower in samples collected over 90 days since antibiotic receipt compared to samples collected while a child was receiving antibiotics (estimate −1.33, *P* = 3 × 10^−4^). Of note, when analyzing antibiotic-free days as a continuous variable, we only identified a significant negative association with *Acinetobacter* abundance after adjustment for covariates (Table S4).

**Fig 2:**
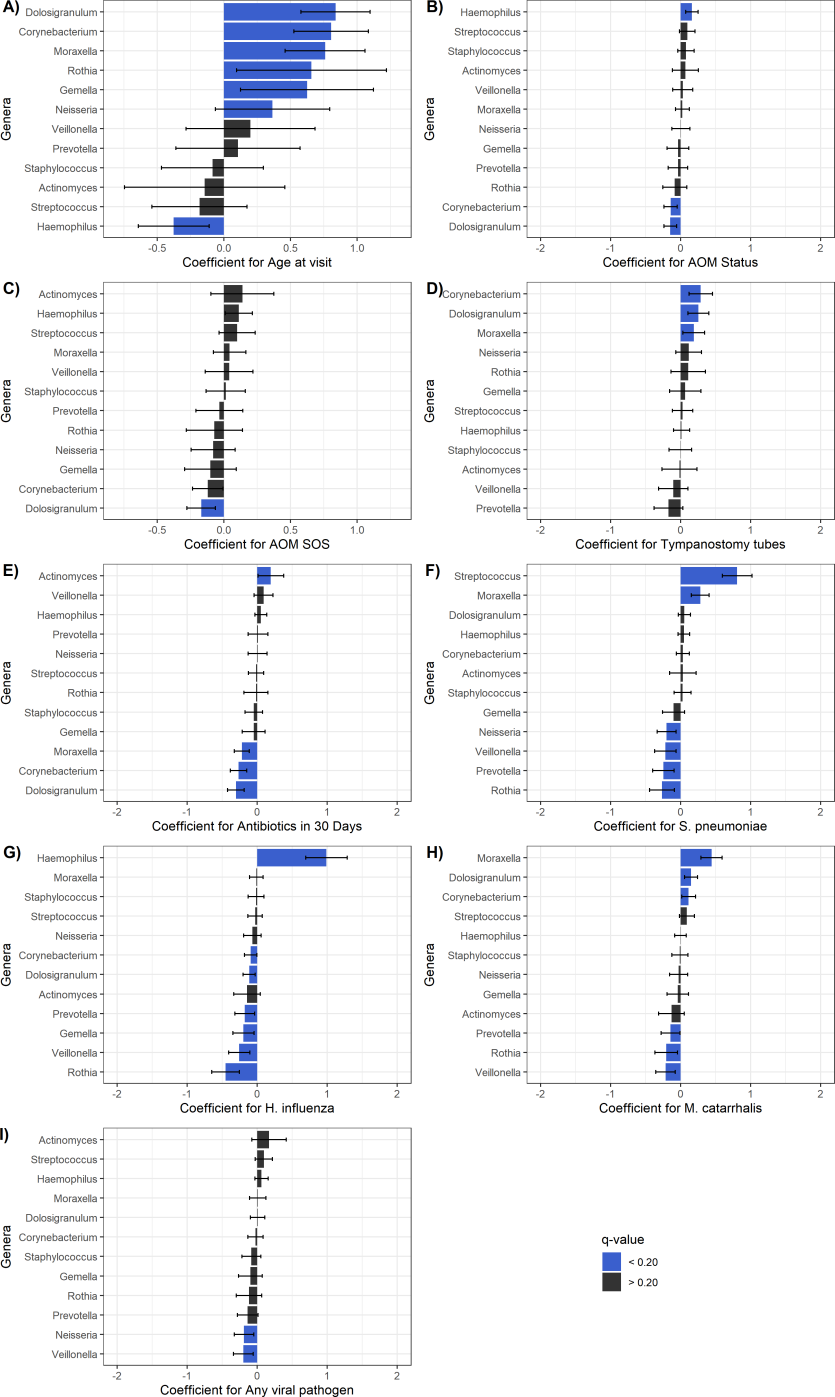
Participant characteristics and exposures associated with changes in the abundance of specific bacterial genera. We fit generalized linear mixed models with CLR-transformed counts evaluating associations between patient characteristics and environmental exposures and the relative abundances of bacterial genera within participant nasopharyngeal samples, with subject included as a random effect. The coefficient and the corresponding 95% confidence interval (CI) are shown for panel **B**, and the logarithm of the odds ratio and the corresponding 95% confidence intervals (on the log scale) are shown for panels **A **and **C**–**L**; these correspond to the relative effect sizes of significant associations (*q*  <  0.20), highlighted in blue. Associations were evaluated for (**A**) age at visit; (**B**) AOM status; (**C**) AOM-SOS score; (**D**) presence of tympanostomy tubes; (**E**) receipt of antibiotics in the past 30 days; (**F**) culture of *S. pneumoniae*; (**G**) culture of *H. influenzae*; (**H**) culture of *M. catarrhalis*; and (**I**) detection of any viral pathogen.

We next sought to identify associations between the relative abundance of specific bacterial genera and various sociodemographic features and exposures at the time of sample collection, including age; season; 30 or fewer antibiotic-free days; presence of a smoker in the participating child’s household; presence of different respiratory viruses; tympanostomy tubes; presence of the three bacterial pathogens; participation in a congregate care setting (i.e., exposure to three or more children for 10 or more hours each week); presence of AOM; and AOM-SOS score (Fig. 2; Fig. S2). Age was the factor most strongly associated with differential abundance of specific genera. We identified positive associations between age and the abundance of *Dolosigranulum*, *Corynebacterium*, *Moraxella*, *Rothia*, *Gemella*, and *Neisseria* ASVs, while age was negatively associated with abundance of *Haemophilus* ASVs. Further, we identified negative associations between the abundance of *Corynebacterium*, *Dolosigranulum*, and *Moraxella* and having 30 or fewer antibiotic-free days prior to sample collection, while abundance of these three genera was positively associated with the presence of tympanostomy tubes. We identified only a few significant associations between the abundance of specific genera and the presence of viral respiratory pathogens. The presence of any virus was negatively associated with the abundance of *Neisseria* and *Veillonella* ASVs. Detection of adenovirus was positively associated with *Haemophilus* ASVs and negatively associated with *Corynebacterium* and *Staphylococcus* ASVs, while detection of parainfluenza was associated with increased abundance of *Dolosigranulum* ASVs and decreased abundance of *Prevotella* ASVs (Fig. 2; Fig. S2). We did not identify any significant associations with EV-HRV, metapneumovirus, or RSV. There were multiple significant associations with bacterial pathogens. The presence of *S. pneumoniae* was positively associated with *Moraxella* ASVs, while both *S. pneumoniae* and *H. influenzae* were negatively associated with *Gemella*, *Neisseria*, *Veillonella*, and *Rothia* ASV abundance. *H. influenzae* was associated with decreased abundance of *Corynebacterium* and *Dolosigranulum* ASVs (Fig. 2; Fig. S2).

### Unsupervised clustering of microbiome profiles identifies associations with participant characteristics, AOM status, and pathogen burden

**Fig 3:**
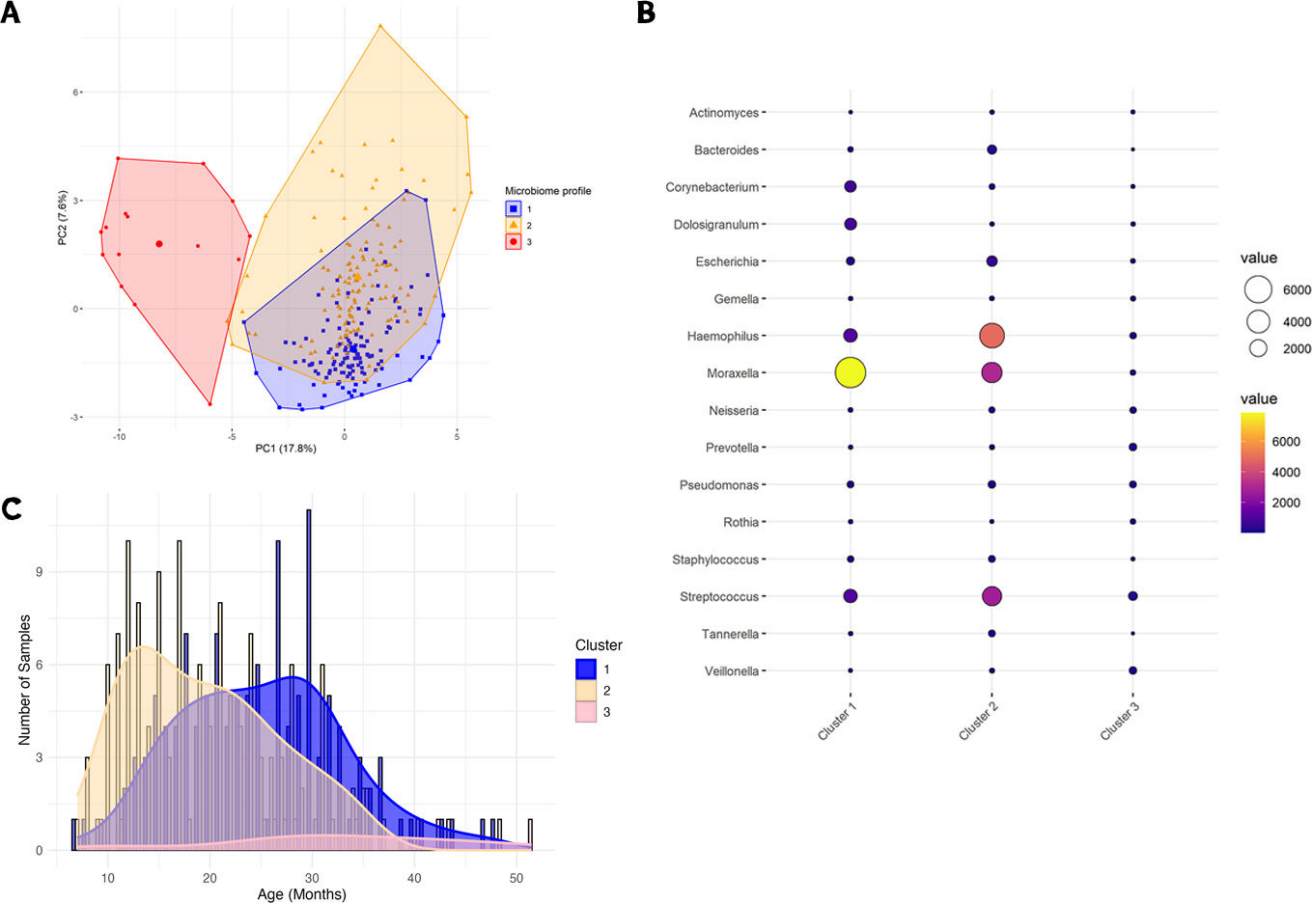
Nasopharyngeal microbiome profiles generated by unsupervised clustering. (**A**) Principal coordinate (PC) plot of Euclidean distances showing clustering of nasopharyngeal samples by microbiome profile. Cluster 1 is shown in blue, Cluster 2 is shown in yellow, and Cluster 3 is shown in red. (**B**) A heat map of mean relative abundances of highly abundant genera within nasopharyngeal samples grouped by cluster. (**C**) Distribution of samples attributed to each cluster by age at time of sample collection.

We used unsupervised clustering to classify nasopharyngeal samples into three distinct microbiome profiles (Table 3, Fig. 3). Cluster 1 (*n* = 125, 46.6%) was characterized by high abundance of *Moraxella* ASVs, while Cluster 2 (*n* = 128, 47.8%) was distinguished by high abundance of *Haemophilus* ASVs. We did not identify increased abundance of specific taxa in Cluster 3 (*n* = 15, 5.6%) (Fig. 3B). We further observed that Cluster 2 was predominant in samples collected from younger children, with the prevalence of Clusters 1 and 3 increasing with age (Fig. 3C). The clusters additionally differed by characteristics at the time of sample collection, including median age (*P* < 1 × 10^−4^), sex (*P* = 0.002), participation in congregate care settings (*P* = 0.007), season of sample collection (*P* < 1 × 10^−3^), AOM-SOS score (*P* = 0.005), number of antibiotic-free days prior to sample collection (*P* < 1 × 10^−4^), and detection of three bacterial pathogens (*S. pneumoniae*: *P* < 1 × 10^−4^; *H. influenzae*: *P* < 1 × 10^−4^; *M. catarrhalis*: *P* = 0.001) (Table 3). We specifically evaluated for associations between these clusters and the presence of AOM at the time of sample collection and found that Cluster 2 had a higher odds of AOM detection compared to Cluster 1 (odds ratio [OR] = 2.63, 95% confidence interval [CI]: 1.45–4.77), but not Cluster 3 (OR = 0.64, 95% CI: 0.94–17.86), though this latter result may be due to the very small number of samples classified as Cluster 3 (Table S5).

**TABLE 3:** Associations between microbiome profiles and clinical characteristics

Sample characteristic	Cluster 1 (*N* = 125, 46.6%)	Cluster 2 (*N* = 128, 47.8%)	Cluster 3 (*N* = 15, 5.6%)	*P* ^*a*^
Age (months), median (IQR)	24 (18, 30)	18.5 (13, 24)	33 (25.5, 40.5)	**<1 × 10^−4^**
Female sex, *n* (%)	33 (26.4%)	59 (46.1%)	8 (53.3%)	**0.002**
Smoker in child’s home, *n* (%)	43 (34.4%)	32 (25%)	1 (6.7%)	**0.041**
Exposed to three or more children for 10+ hours/week	98 (78.4%)	118 (92.2%)	13 (86.7%)	**0.007**
Season				
January through April, *n* (%)	25 (20.0%)	50 (39.1%)	3 (20%)	**<1 × 10^−3^**
May through September, *n* (%)	57 (45.6%)	56 (43.8%)	5 (33.3%)	
October through December, *n* (%)	43 (34.4%)	22 (17.2%)	7 (46.7%)	
Tympanostomy tubes placed, *n* (%)	65 (52.0%)	56 (43.8%)	8 (53.3%)	0.387
AOM status, *n* (%)				
AOM	33 (26.4%)	59 (46.1%)	3 (20.0%)	**0.002**
No AOM	92 (73.6%)	69 (53.9%)	12 (80.0%)	
AOM symptom score, median (IQR)	1 (0, 3)	2 (0, 6)	1 (0, 2)	**0.005**
Currently taking antibiotics, *n* (%)	7 (5.6%)	16 (12.5%)	1 (6.7%)	0.153
Antibiotic-free days, median (IQR)	144 (83, 300)	52 (13.5, 135.5)	244 (81, 384)	**<1 × 10^−4^**
Bacterial pathogens detected				
*S. pneumoniae*, *n* (%)	58 (46.4%)	74 (57.8%)	0 (0%)	**<1 × 10^−4^**
*H. influenzae*, *n* (%)	31 (24.8%)	88 (68.8%)	1 (6.7%)	**<1 × 10^−4^**
*M. catarrhalis*, *n* (%)	84 (67.2%)	71 (55.5%)	3 (20.0%)	**0.001**
Respiratory viruses detected				
Influenza A/B/C, *n* (%)	7 (5.6%)	8 (6.2%)	1 (6.7%)	1
Metapneumovirus, *n* (%)	11 (8.8%)	15 (11.7%)	0 (0%)	0.429
RSV, *n* (%)	11 (8.8%)	13 (10.2%)	0 (0%)	0.568
Enterovirus/human rhinovirus, *n* (%)	72 (57.6%)	87 (68%)	5 (33.3%)	**0.019**
Parainfluenza virus, *n* (%)	27 (21.6%)	17 (13.3%)	1 (6.7%)	0.137
Human adenovirus, *n* (%)	26 (20.8%)	47 (36.7%)	3 (20.0%)	**0.015**

^*a*^ *P *< 0.05 was considered to be statistically significant.

### Identification of exposures associated with microbiome profile transitions

Because we found that the Cluster 2 microbiome profile had higher odds of AOM detection, we sought to further characterize transitions between microbiome profiles, including identifying events that precipitated a change in microbiome cluster for each child (Fig. S3). Out of 182 samples, we identified 86 microbiome profile transitions that occurred between samples collected consecutively from the same child. Given that the odds of AOM detection were higher among children with samples classified as Cluster 2, we sought to identify exposures associated with transitions from Clusters 1 and 3 to Cluster 2. Specifically, we evaluated events occurring between two visits, including increasing age, antibiotic use, and surgery for tympanostomy tube placement (Table 4). Increasing age was associated with a decreased likelihood of transitioning from Clusters 1 or 3 to Cluster 2 (OR = 0.938, 95% CI: 0.882–0.998) and an increased likelihood of transitioning from Cluster 2 to Clusters 1 or 3 (OR = 1.09, 95% CI: 1.01–1.18). Antibiotic use preceding sample collection was strongly associated with a transition from Clusters 1 or 3 to Cluster 2 (OR = 3.07, 95% CI: 1.34–7.00). We did not identify any significant associations between tympanostomy tube placement and microbiome profile transitions.

**TABLE 4:** Associations between exposures prior to sample collection and odds of a transition between microbiome profiles

Cluster transition	Exposure since last visit	OR	Standard error	Statistic	*P* ^*a*^	95% CI
Other to Cluster 2	Antibiotic use	3.07	1.29	2.66	**0.008**	1.34–7.00
Other to Cluster 2	Increasing age	0.938	0.030	−2.03	**0.042**	0.882–0.998
Other to Cluster 2	Tympanostomy tube placement	1.77	0.748	1.35	0.178	0.772–4.05
Cluster 2 to Other	Antibiotic use	0.75	0.428	−0.504	0.614	0.245–2.3
Cluster 2 to Other	Increasing age	1.09	0.043	2.24	**0.025**	1.01–1.18
Cluster 2 to Other	Tympanostomy tube placement	0.477	0.264	−1.34	0.181	0.162–1.41

^*a*^ Associations between cluster transition and antibiotic use, increasing age, and tympanostomy tube placement were evaluated in a single mixed effects logistic regression model, adjusting for each exposure. *P* < 0.05 was considered to be statistically significant.

### AOM and antibiotic exposures are associated with changes in the relative abundance of specific taxa

Given the associations between cluster transitions with age, antibiotic receipt, and AOM, we evaluated the change in relative abundance of specific taxa that have previously been associated with AOM or respiratory health (Table S6). After adjusting for age (associations with age shown in Table S7), we found that AOM episodes were significantly associated with decreased abundance of *Corynebacterium* (estimate: −0.8087, *P* = 0.032) and *Dolosigranulum* (estimate: −0.9479, *P* = 0.016), but increased abundance of *Haemophilus* ASVs (estimate: 1.4728, *P* = 0.0006). We did not identify any significant associations between AOM and relative abundance of *Moraxella* or *Streptococcus* ASVs; however, *Moraxella* (estimate: 0.0047, *P* = 0.0005) and *Streptococcus* (estimate: 0.0029, *P* = 0.018) ASVs were positively associated with the number of antibiotic-free days prior to sample collection.

## DISCUSSION

There is increasing evidence that the URT microbiome plays a pivotal role in acute respiratory infections, including AOM. We therefore sought to evaluate associations between URT microbiome features and participant characteristics and exposures in a cohort of children with rAOM. We found that age was strongly associated both with microbiome composition and the occurrence of AOM, regardless of whether children had tympanostomy tubes at a given visit. Further, age was positively associated with the abundance of genera linked to respiratory health (i.e., *Dolosigranulum* and *Corynebacterium*), while the presence of AOM or recent exposure to antibiotics was associated with a lower abundance of these genera. Moreover, increasing age was associated with a transition to microbiome profiles characterized by lower AOM incidence and bacterial otopathogen burden, while antibiotic use preceding sample collection was associated with transition to a cluster associated with greater AOM incidence at a subsequent visit. These findings indicate that microbiome alterations associated with aging may contribute to decreased AOM incidence as children age, while systemic antibiotic use may induce dysbiosis, thereby enhancing AOM susceptibility.

Prior studies have demonstrated that colonization with pathobionts in early life, including *S. pneumoniae*, *M. catarrhalis*, and non-typeable *H. influenzae*, is associated with later risk of AOM (14, 46–48). In a longitudinal cohort study of 358 respiratory illness-prone and non-prone/healthy children, Chapman and colleagues found that half of respiratory illness-prone children were colonized by *S. pneumoniae*, *M. catarrhalis*, and non-typeable *H. influenzae* by 15.6 months of age, while only 34% of the healthy comparator group was colonized by 40 months of age (14). We and others have demonstrated that the URT microbiome influences colonization susceptibility for multiple respiratory pathogens and that microbiome composition influences the likelihood of pathogen colonization (17, 49–51). In the current study, we found that children in this cohort were frequently colonized by *S. pneumoniae*, *H. influenzae,* and *M. catarrhalis*, though only *S. pneumoniae* and *H. influenzae* were more frequently detected in samples collected during AOM episodes compared to AOM-free visits. The high prevalence of these pathogens regardless of AOM status suggests that other factors contribute to the development of an AOM episode.

AOM has long been thought to be a polymicrobial infection wherein respiratory viruses trigger inflammation that drives bacterial pathogens to the middle ear, and previous studies have identified multiple respiratory viruses in nasopharyngeal swabs and middle ear fluid from children with AOM (25, 52–55). Respiratory viruses were frequently detected in our cohort, with nearly 80% of samples testing positive for one or more respiratory viruses; however, respiratory virus detection was only slightly more frequent in samples collected during an AOM episode, and we did not identify any significant differences among specific viruses between AOM and non-AOM samples. Notably, a recent study in the PREVAIL cohort identified prolonged viral shedding, defined as a persistently positive PCR test for four or more weeks, in nearly a quarter of viral respiratory infections (56). These findings suggest that it is difficult to identify active viral infections using PCR, and further studies are needed to define the contribution of respiratory viruses to rAOM. Additionally, the impacts of respiratory viruses on the microbiome may also depend on the characteristics of the infection, as Chonmaitree and colleagues found that only symptomatic respiratory viral infections resulted in significant increases in otopathogen abundance and decreases in respiratory commensals, including *Dolosigranulum* and *Corynebacterium* (23). Additional studies are needed to understand how respiratory viruses contribute to rAOM. Finally, new approaches and studies are needed to assess the role of the virome and mycobiome in rAOM, as both kingdoms have been underexplored within the URT.

Children with rAOM are subject to frequent antibiotic exposures, which can directly impact the composition of the microbiome across multiple body sites, including the gut and respiratory tract. Importantly, antibiotic exposures can have lasting impacts on microbiome composition and have been previously associated with the development of asthma and the frequency of respiratory infections later in childhood (19, 57). In a chinchilla model of AOM, Duff and colleagues demonstrated that antibiotic treatment led to an increased abundance of opportunistic pathogens and decreased abundance of commensal species within the nasopharynx (58). Interestingly, this effect was observed whether the antibiotics were administered orally or via tympanostomy tubes. There have been similar observations among infant cohorts, wherein antibiotic exposures in the 6 months prior to sample collection were associated with increased abundance of *Haemophilus*, decreased abundance of *Corynebacterium* and *Dolosigranulum*, and a greater likelihood of having an AOM episode at the time of sample collection (15). There is increasing evidence that antibiotic exposures directly alter microbiome maturation in early childhood (59–61). Using longitudinal URT microbiome samples collected from a large birth cohort study, Raita and colleagues modeled the relationship between specific bacterial genera and age but found that the age discriminatory taxa differed according to antibiotic exposure (60). Among children without antibiotic exposures, *Dolosigranulum* increased with age; however, among those with previous antibiotic exposures, *Haemophilus* increased with age, indicating that antibiotics resulted in altered maturation patterns that may contribute to development of rAOM.

We and others have previously observed strong correlations between age and URT microbiome composition, particularly during infancy, childhood, and early adolescence (62–65). Hernandez-Leyva and colleagues found that age was the factor most predictive of nasopharyngeal microbiome composition (65). Further, Stearns and colleagues found that age-associated shifts in the URT microbiome were most pronounced in early childhood, the same period during which children are most at risk of AOM and other respiratory infections (66). We previously found that age-associated changes in URT microbiome composition distinguished children and adolescents with symptomatic COVID-19 infections from those with asymptomatic infection, suggesting that microbiome composition likely underlies associations between age and other respiratory illnesses such as AOM (62). In the current study, we similarly found that microbiome composition was strongly influenced by age, with children in our cohort shifting from microbiome profiles with greater abundance of *Haemophilus*, *Streptococcus*, and *Moraxella* to a *Moraxella*-dominated microbiome or more diverse microbiome over the course of the study. We further observed that antibiotic receipt prior to sample collection was associated with a shift back to the *Haemophilus*, *Streptococcus*, and *Moraxella*-dominated microbiome profile that was both more common among younger children and associated with the presence of AOM. We did not observe any significant associations between tympanostomy tube placement and shifts in overall microbiome composition, though children with tympanostomy tubes did have greater abundances of *Corynebacterium*, *Dolosigranulum*, and *Moraxella* compared to children without tympanostomy tubes. Notably, children with tympanostomy tubes were significantly older at post-placement visits than children without tympanostomy tubes. Thus, the increased abundances of these taxa are most likely due to increasing age. We previously found that tympanostomy tube placement did not lead to a decrease in subsequent AOM episodes compared to medical management (9). Based on the current study, tympanostomy tubes do not impact microbiome compositions, and it is possible that children with rAOM will continue to have AOM/otorrhea episodes until their microbiome has matured. Taken together, these findings suggest that AOM may be a disease of URT microbiome immaturity, in which antibiotics and respiratory illnesses hinder the development of a more mature state that is resilient to otopathogen colonization. Moreover, this finding may also help explain our previous observation that tympanostomy tubes do not reduce the number of AOM episodes in this population.

Our study had several strengths and limitations. Strengths included the longitudinal nature of the cohort, including serial sampling and detailed clinical characterization. Moreover, this is one of the few studies that specifically evaluated children with well-characterized AOM histories and sample collection during periods of health and during AOM episodes. Limitations of the study included the lack of a healthy comparator group and the use of 16S rRNA sequencing, which precluded species-level identification. There is a chance of misidentification of cultured pathogens using the Vitek 2 system. Thus, some samples may be misclassified regarding the presence of cultured otopathogens. Future longitudinal studies will be needed to compare the development of the URT microbiome in healthy children and those who eventually develop AOM. Our study did not evaluate the role of fungal species or of viruses outside of the specific respiratory viral pathogens that we detected using targeted PCR-based assays. Further, we did not specifically evaluate for other otopathogens such as *Streptococcus pyogenes* or *Staphylococcus aureus*. New approaches and additional studies are needed to enhance our ability to conduct shotgun metagenomic sequencing in low biomass samples and to evaluate fungal and viral species within the URT. Finally, respiratory samples were collected in Amies liquid media, which may not be as stable as other media (i.e., viral transport media) with respect to viral longevity. Thus, this analysis may have relatively lower rates of viral detection.

Our study provides one of the first longitudinal evaluations of the URT microbiome among children with rAOM. We found that AOM was strongly associated with younger age. In turn, age was the strongest factor associated with microbiome composition, and increasing age was associated with shifts to microbiome profiles that were less prevalent among children with an AOM episode and that were not dominated by taxa that include common otopathogens. Finally, we observed that systemic antibiotic exposures to treat AOM were associated with shifts in microbiome composition and transitions to microbiome profiles that are both dominated by bacterial pathogens. These findings underscore the importance of the microbiome in rAOM pathogenesis and suggest that microbiome-targeted therapeutics could potentially be used to promote microbiome maturation and/or recovery.

## Data Availability

The sequencing data set supporting the conclusions of this study is available in the Sequence Read Archive (PRJNA1327964). The metadata associated with this study are available at https://github.com/YJulyXing/Recurrent_acute_otitis_media_nasopharyngeal_samples_repo.

## SUPPLEMENTAL MATERIAL

Supplemental Tables and Figures msphere.00468-25-s0001.docx Tables S1-S7 and Figures S1-S3.

ASM does not own the copyrights to Supplemental Material that may be linked to, or accessed through, an article. The authors have granted ASM a non-exclusive, world-wide license to publish the Supplemental Material files. Please contact the corresponding author directly for reuse.
