# Supplementary material for: Age and antibiotic use influence longitudinal dynamics of the upper respiratory microbiome in children with recurrent acute otitis media
Source: mSphere. 2025 Nov 12;10(12):e00468-25. doi: 10.1128/msphere.00468-25 (PMC12724347; doi:10.1128/msphere.00468-25)
Supplement: Supplemental Tables and Figures — Tables S1-S7 and Figures S1-S3. [file msphere.00468-25-s0001.docx]

**Supplemental Tables**

| **Supplemental Table 1.** Prevalence of respiratory pathogens by season | | | |
| --- | --- | --- | --- |
| **Respiratory pathogens** | **January – April**  **(% positive samples)** | **May – September**  **(% positive samples)** | **October – December**  **(% positive samples)** |
| **Viral pathogens** | | | |
| Human adenovirus | 42% | 26% | 17% |
| Influenza A | 3% | 0% | 0% |
| Influenza B | 14% | 3% | 1% |
| Influenza C | 9% | 2% | 1% |
| Enterovirus/human rhinovirus | 58% | 67% | 56% |
| Human metapneumovirus | 14% | 8% | 7% |
| Parainfluenza | 10% | 19% | 19% |
| Respiratory syncytial virus | 21% | 3% | 7% |
| Any virus detected | 83% | 81% | 72% |
| **Bacterial pathogens** | | | |
| Group A Streptococcus | 10% | 3% | 4% |
| *H. influenzae* | 54% | 47% | 31% |
| *M. catarrhalis* | 67% | 49% | 67% |
| *S. pneumoniae* | 47% | 52% | 47% |

| **Supplemental Table 2.** Correlations between the relative abundance of specific genera within participant nasopharyngeal samples and antibiotic-free days | | | |
| --- | --- | --- | --- |
| **Genus** | **r** | **P value** | **Adjusted P value*** |
| *Acinetobacter* | -0.1273 | 0.0376 | 0.1506 |
| *Corynebacterium* | 0.2628 | <1x10^-4^ | **1x10^-4^** |
| *Delftia* | -0.1233 | 0.0441 | 0.1506 |
| *Dolosigranulum* | 0.3175 | <1x10^-4^ | **<1x10^-4^** |
| *Escherichia* | -0.2658 | <1x10^-4^ | **1x10^-4^** |
| *Fusobacterium* | 0.1285 | 0.0359 | 0.1506 |
| *Gemella* | 0.1231 | 0.0445 | 0.1506 |
| *Granulicatella* | 0.1307 | 0.0328 | 0.1506 |
| *Haemophilus* | -0.1645 | 0.0071 | 0.0565 |
| *Moraxella* | 0.355 | <1x10^-4^ | **<1x10^-4^** |
| *Pseudomonas* | -0.1227 | 0.0452 | 0.1506 |
| *Ralstonia* | -0.133 | 0.0298 | 0.1506 |
| **Adjusted p-values <0.05 were considered significant.* | | | |

| **Supplemental Table 3.** Adjusted model of associations between relative abundance of specific genera within participant nasopharyngeal samples and antibiotic-free days as a categorical variable | | | | | | |
| --- | --- | --- | --- | --- | --- | --- |
| **Genus** | **Reference** | **Test** | **Estimate** | **Std Error** | **P-value** | **Adjusted p-value*** |
| *Burkholderia* | Currently taking antibiotics | 30-60 days prior to sample collection | -1.621 | 0.5724 | 0.0051 | 0.0906 |
| *Burkholderia* | Currently taking antibiotics | 60-90 days prior to sample collection | -1.6603 | 0.6366 | 0.0098 | 0.1312 |
| *Burkholderia* | Currently taking antibiotics | More than 90 days prior to sample collection | -1.2546 | 0.5215 | 0.0171 | 0.1826 |
| *Burkholderia* | 30-60 days prior to sample collection | Currently taking antibiotics | 1.621 | 0.5724 | 0.0051 | 0.1643 |
| *Corynebacterium* | Currently taking antibiotics | 30-60 days prior to sample collection | 2.322 | 0.731 | 0.0017 | 0.0558 |
| *Corynebacterium* | Currently taking antibiotics | 60-90 days prior to sample collection | 2.2727 | 0.812 | 0.0057 | 0.0906 |
| *Corynebacterium* | Currently taking antibiotics | More than 90 days prior to sample collection | 1.7778 | 0.6681 | 0.0085 | 0.1232 |
| *Corynebacterium* | 30-60 days prior to sample collection | Currently taking antibiotics | -2.322 | 0.731 | 0.0017 | 0.1395 |
| *Corynebacterium* | More than 90 days prior to sample collection | Currently taking antibiotics | -1.7778 | 0.6681 | 0.0085 | 0.1694 |
| *Dolosigranulum* | Currently taking antibiotics | More than 90 days prior to sample collection | 1.711 | 0.6882 | 0.0138 | 0.1576 |
| *Dolosigranulum* | Antibiotics less than 30 days prior | 30-60 days prior to sample collection | 1.9144 | 0.6734 | 0.005 | 0.1326 |
| *Dolosigranulum* | Antibiotics less than 30 days prior | More than 90 days prior to sample collection | 2.1062 | 0.6445 | 0.0013 | 0.0687 |
| *Dolosigranulum* | 30-60 days prior to sample collection | Antibiotics less than 30 days prior | -1.9144 | 0.6734 | 0.005 | 0.1643 |
| *Dolosigranulum* | More than 90 days prior to sample collection | Antibiotics less than 30 days prior | -2.1062 | 0.6445 | 0.0013 | 0.0597 |
| *Escherichia* | Currently taking antibiotics | Antibiotics less than 30 days prior | -0.9759 | 0.3068 | 0.0017 | 0.0558 |
| *Escherichia* | Currently taking antibiotics | 60-90 days prior to sample collection | -1.098 | 0.3283 | 0.001 | 0.053 |
| ***Escherichia*** | **Currently taking antibiotics** | **More than 90 days prior to sample collection** | **-1.33** | **0.2719** | **0** | **3x10^-4^** |
| *Escherichia* | Antibiotics less than 30 days prior | Currently taking antibiotics | 0.9759 | 0.3068 | 0.0017 | 0.0688 |
| *Escherichia* | 30-60 days prior to sample collection | More than 90 days prior to sample collection | -0.6729 | 0.2322 | 0.0042 | 0.1643 |
| *Escherichia* | 60-90 days prior to sample collection | Currently taking antibiotics | 1.098 | 0.3283 | 0.001 | 0.1591 |
| *Escherichia* | More than 90 days prior to sample collection | Currently taking antibiotics | 1.33 | 0.2719 | 0 | 3x10^-4^ |
| *Escherichia* | More than 90 days prior to sample collection | 30-60 days prior to sample collection | 0.6729 | 0.2322 | 0.0042 | 0.096 |
| *Gemella* | Currently taking antibiotics | 60-90 days prior to sample collection | 1.3197 | 0.5255 | 0.0129 | 0.1576 |
| *Lachnospiraceae [G-2]* | Currently taking antibiotics | Antibiotics less than 30 days prior | 1.6348 | 0.4717 | 7.00E-04 | 0.0524 |
| *Lachnospiraceae [G-2]* | Antibiotics less than 30 days prior | Currently taking antibiotics | -1.6348 | 0.4717 | 7.00E-04 | 0.0524 |
| *Moraxella* | Currently taking antibiotics | More than 90 days prior to sample collection | 1.732 | 0.5971 | 0.0042 | 0.0906 |
| ***Moraxella*** | **Antibiotics less than 30 days prior** | **More than 90 days prior to sample collection** | **2.4108** | **0.5609** | **0** | **0.0044** |
| *Moraxella* | 30-60 days prior to sample collection | More than 90 days prior to sample collection | 1.6519 | 0.5124 | 0.0015 | 0.1395 |
| *Moraxella* | More than 90 days prior to sample collection | Currently taking antibiotics | -1.732 | 0.5971 | 0.0042 | 0.096 |
| ***Moraxella*** | **More than 90 days prior to sample collection** | **Antibiotics less than 30 days prior** | **-2.4108** | **0.5609** | **0** | **0.0022** |
| *Moraxella* | More than 90 days prior to sample collection | 30-60 days prior to sample collection | -1.6519 | 0.5124 | 0.0015 | 0.0597 |
| *Neisseriaceae [G-1]* | Currently taking antibiotics | Antibiotics less than 30 days prior | -1.5298 | 0.5331 | 0.0046 | 0.0906 |
| *Neisseriaceae [G-1]* | Antibiotics less than 30 days prior | Currently taking antibiotics | 1.5298 | 0.5331 | 0.0046 | 0.1326 |
| *Pseudomonas* | Currently taking antibiotics | More than 90 days prior to sample collection | -1.6464 | 0.5324 | 0.0023 | 0.0611 |
| *Pseudomonas* | More than 90 days prior to sample collection | Currently taking antibiotics | 1.6464 | 0.5324 | 0.0023 | 0.0733 |
| *Veillonella* | Antibiotics less than 30 days prior | 30-60 days prior to sample collection | -1.184 | 0.4464 | 0.0087 | 0.1984 |
| *Results are from a mixed model with random effects evaluating for the association between the abundance of specific taxa and time since antibiotic exposure as a categorical level. Covariates in the model included clinical status (AOM vs. no AOM); exposure to congregate care; smoker in child’s home; season; presence of tympanostomy tubes; age at time of sample collection; AOM-SOS score at time of sample collection; and the detection of* S. pneumoniae, H. influenzae, M. catarrhalis*, influenza A, B, and/or C, human metapneumovirus, respiratory syncytial virus, enterovirus/human rhinovirus, parainfluenza, and/or adenovirus. Results with a p-value <0.2 are shown.*  **Adjusted p-values <0.05 were considered significant.* | | | | | | |

| **Supplemental Table 4.** Adjusted model of associations between relative abundance of specific genera within participant nasopharyngeal samples and antibiotic-free days as a continuous variable | | | | |
| --- | --- | --- | --- | --- |
| **Genus** | **Value** | **Std Error** | **P-value** | **Adjusted p-value*** |
| *Acinetobacter* | -0.0031 | 9x10^-4^ | 4x10^-4^ | **0.016** |
| *Moraxella* | 0.0036 | 0.0013 | 0.0044 | 0.0885 |
| *Results are from a mixed model with random effects evaluating for the association between the abundance of specific taxa and time since antibiotic exposure as a continuous level. Covariates in the model included clinical status (AOM vs. no AOM); exposure to congregate care; smoker in child’s home; season; presence of tympanostomy tubes; age at time of sample collection; AOM-SOS score at time of sample collection; and the detection of* S. pneumoniae, H. influenzae, M. catarrhalis*, influenza A, B, and/or C, human metapneumovirus, respiratory syncytial virus, enterovirus/human rhinovirus, parainfluenza, and/or adenovirus. Results with a p-value <0.2 are shown.*  **Adjusted p-values <0.05 were considered significant.* | | | | |

| **Supplemental Table 5.** Odds of AOM detection in children with URT microbiome samples classified into different clusters | | | | |
| --- | --- | --- | --- | --- |
| Reference | Test | OR | 95% CI | P-value* |
| Cluster1 | Cluster2 | 2.6337 | 1.4529 – 7.7742 | **0.0014** |
| Cluster1 | Cluster3 | 0.6428 | 0.1483 – 2.7863 | 0.5548 |
| Cluster3 | Cluster2 | 4.0976 | 0.9399 – 17.8644 | 0.0605 |
| **Adjusted p-values <0.05 were considered significant.* | | | | |

| **Supplemental Table 6.** Association between the abundance of specific taxa and number of antibiotic-free days or presence of AOM at time of sample collection, adjusted for age | | | | | | |
| --- | --- | --- | --- | --- | --- | --- |
| Genus | Exposure | Estimate | Standard error | Statistic | Degrees of freedom | P-value* |
| *Corynebacterium* | AOM | -0.8087 | 0.3760 | -2.1506 | 265.88 | **0.032** |
| *Dolosigranulum* | AOM | -0.9479 | 0.3923 | -2.416 | 266.46 | **0.016** |
| *Haemophilus* | AOM | 1.4728 | 0.4281 | 3.440 | 263.91 | **0.0006** |
| *Moraxella* | AOM | 0.4233 | 0.3629 | 1.1665 | 261.23 | 0.2444 |
| *Streptococcus* | AOM | 0.4578 | 0.3324 | 1.3772 | 266.60 | 0.169 |
| *Corynebacterium* | Antibiotic-free days | 0.0012 | 0.0014 | 0.8182 | 197.91 | 0.414 |
| *Dolosigranulum* | Antibiotic-free days | 0.0023 | 0.0014 | 1.5782 | 187.37 | 0.116 |
| *Haemophilus* | Antibiotic-free days | -0.0014 | 0.0016 | -0.8592 | 206.51 | 0.391 |
| *Moraxella* | Antibiotic-free days | 0.0047 | 0.0013 | 3.5312 | 189.84 | **0.0005** |
| *Streptococcus* | Antibiotic-free days | 0.0029 | 0.0012 | 2.3791 | 207.80 | **0.018** |
| **Adjusted p-values <0.05 were considered significant.* | | | | | | |

| **Supplemental Table 7.** Association between age at time of sample collection and the abundance of specific taxa | | | | | | | |
| --- | --- | --- | --- | --- | --- | --- | --- |
| Genus | Effect | Term | estimate | Standard error | Statistic | Degrees of freedom | P-value* |
| *Pseudomonas* | fixed | Age | -0.0851 | 0.02181 | -3.9034 | 164.72 | **0.0001** |
| *Corynebacterium* | fixed | Age | 0.0914 | 0.02689 | 3.3981 | 168.84 | **0.0008** |
| *Lactobacillus* | fixed | Age | -0.0425 | 0.0134 | -3.1647 | 166.15 | **0.0018** |
| *Ralstonia* | fixed | Age | -0.0483 | 0.0158 | -3.0519 | 170.75 | **0.0026** |
| *Moraxella* | fixed | Age | 0.0779 | 0.0265 | 2.9428 | 157.37 | **0.0037** |
| *Granulicatella* | fixed | Age | 0.0408 | 0.0154 | 2.6514 | 171.24 | **0.0087** |
| *Dolosigranulum* | fixed | Age | 0.0750 | 0.0289 | 2.5972 | 164.38 | **0.0103** |
| *Rothia* | fixed | Age | 0.0377 | 0.0161 | 2.3495 | 172.01 | **0.0199** |
| *Leptotrichia* | fixed | Age | 0.0295 | 0.0126 | 2.3434 | 157.38 | **0.0204** |
| *Gemella* | fixed | Age | 0.0381 | 0.0166 | 2.2977 | 171.21 | **0.0228** |
| *Campylobacter* | fixed | Age | 0.0238 | 0.0108 | 2.2048 | 169.36 | **0.0288** |
| *Acinetobacter* | fixed | Age | -0.0341 | 0.0160 | -2.1362 | 148.86 | **0.0343** |
| *Fusobacterium* | fixed | Age | 0.0327 | 0.0156 | 2.0901 | 173.00 | **0.0381** |
| *Neisseriaceae.G.1.* | fixed | Age | -0.0386 | 0.0190 | -2.0343 | 169.17 | **0.0434** |
| *Sphingomonas* | fixed | Age | -0.0270 | 0.0135 | -2.0071 | 162.33 | **0.0464** |
| *Tannerella* | fixed | Age | -0.0317 | 0.0187 | -1.6976 | 160.53 | 0.0915 |
| *Haemophilus* | fixed | Age | -0.0408 | 0.0305 | -1.3357 | 164.96 | 0.1835 |
| **Adjusted p-values <0.05 were considered significant.* | | | | | | | |

**Supplemental Figures**


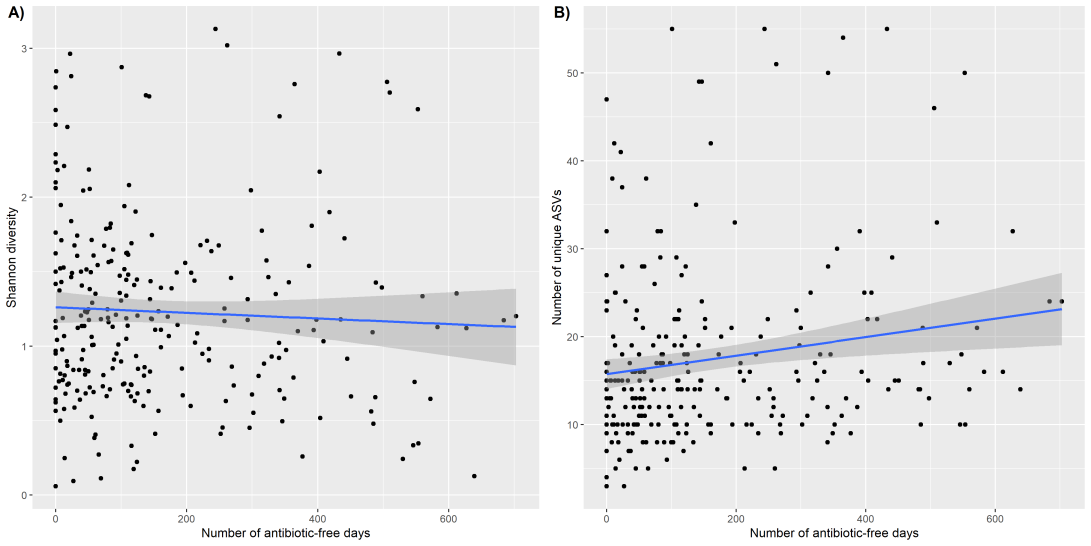


**Supplemental Figure 1.** Nasopharyngeal microbiome alpha diversity and richness by number of antibiotic-free days. Shannon diversity (**A**) and the number of unique ASVs (**B**) are shown by number of antibiotic-free days. Each point represents an individual sample, and the blue lines correspond to a linear model of the relationship between antibiotic-free days and the diversity indices.

**
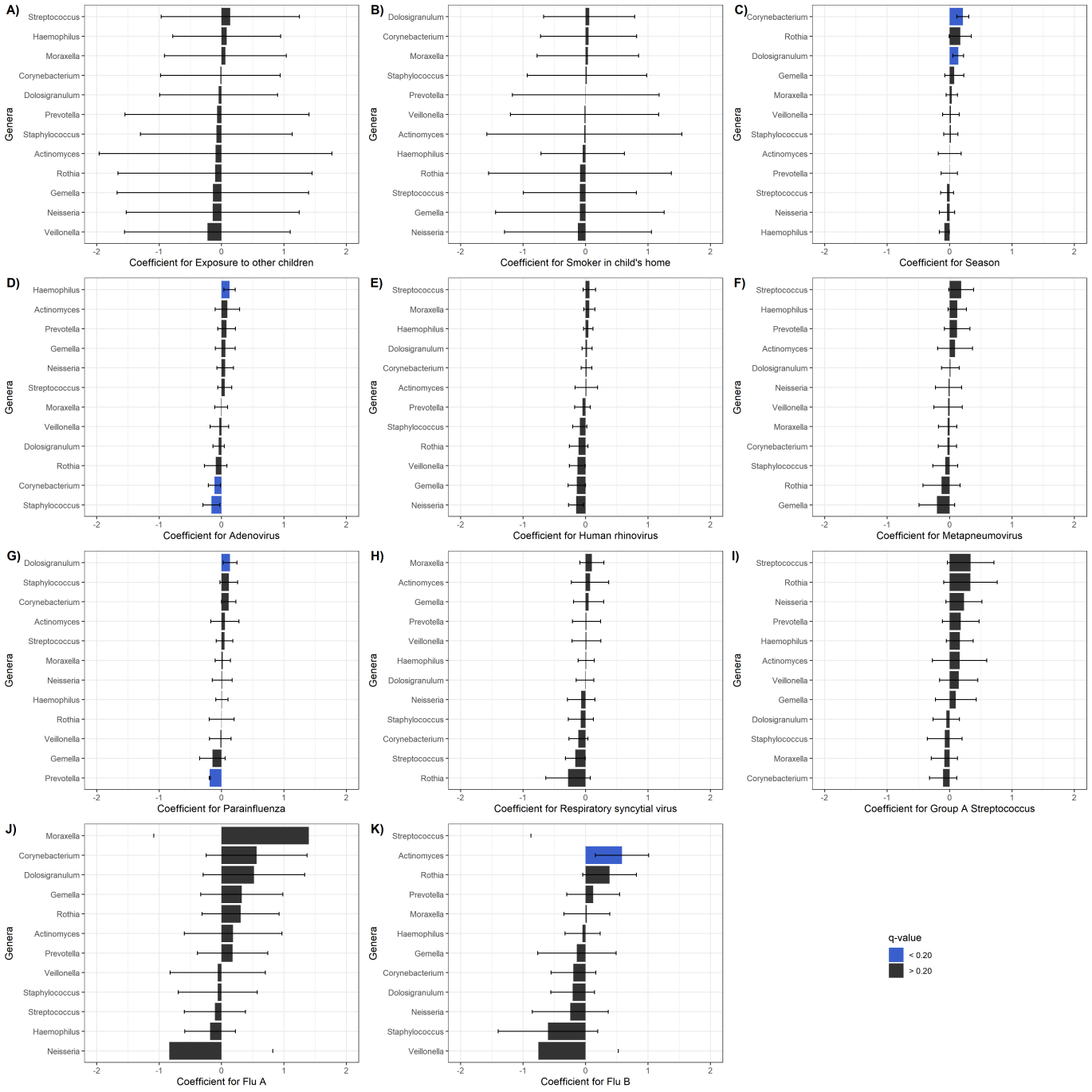
**

**Supplemental Figure 2.** Participant characteristics and exposures associated with changes in the abundance of specific bacterial genera. We fit generalized linear mixed models with CLR-transformed counts evaluating associations between patient characteristics and environmental exposures and the relative abundances of bacterial genera within participant nasopharyngeal samples, with subject included as a random effect. The coefficient and the corresponding 95% confidence interval for (A), and the logarithm of the odds ratio and the corresponding 95% confidence intervals (on the log scale) for (B)-(H), which correspond to the relative effect sizes of associations, are shown for significant associations (*q* < 0.20) in blue. We evaluated associations with **A**) exposure to other children; **B**) smoker in child's home; **C**) season; **D**) detection of adenovirus; **E**) detection of enterovirus/human rhinovirus; **F**) detection of metapneumovirus; **G**) detection of parainfluenza; **H**) detection of respiratory syncytial virus; **I**) detection of group A streptococcus; **J**) detection of influenza A (Flu A); **K**) detection of influenza B (Flu B).

**Supplemental Figure 3.** Longitudinal cluster transitions among individual study participants. Each color indicates the nasopharyngeal microbiome profile identified in the sample collected at each visit. The timing of infections, tympanostomy tube placements, and antibiotic receipt are indicated for each participating child, plotted against their age at the time of each event and/or study visit.
